# Brain-Computer Interface Coupled to a Robotic Hand Orthosis for Stroke Patients’ Neurorehabilitation: A Crossover Feasibility Study

**DOI:** 10.3389/fnhum.2021.656975

**Published:** 2021-06-07

**Authors:** Jessica Cantillo-Negrete, Ruben I. Carino-Escobar, Paul Carrillo-Mora, Marlene A. Rodriguez-Barragan, Claudia Hernandez-Arenas, Jimena Quinzaños-Fresnedo, Isauro R. Hernandez-Sanchez, Marlene A. Galicia-Alvarado, Adan Miguel-Puga, Oscar Arias-Carrion

**Affiliations:** ^1^Division of Research in Medical Engineering, Instituto Nacional de Rehabilitación “Luis Guillermo Ibarra Ibarra,” Mexico City, Mexico; ^2^Neuroscience Division, Instituto Nacional de Rehabilitación “Luis Guillermo Ibarra Ibarra,” Mexico City, Mexico; ^3^Division of Neurological Rehabilitation, Instituto Nacional de Rehabilitación “Luis Guillermo Ibarra Ibarra,” Mexico City, Mexico; ^4^Department of Electrodiagnostic, Instituto Nacional de Rehabilitación “Luis Guillermo Ibarra Ibarra,” Mexico City, Mexico; ^5^Unidad de Trastornos de Movimiento y Sueño (TMS), Hospital General “Dr. Manuel Gea González,” Mexico City, Mexico; ^6^Centro de Innovación Médica Aplicada (CIMA), Hospital General “Dr. Manuel Gea González,” Mexico City, Mexico

**Keywords:** electroencephalography, Fugl-Meyer, hemiparesis, motor intention, TMS, ARAT

## Abstract

Brain-Computer Interfaces (BCI) coupled to robotic assistive devices have shown promise for the rehabilitation of stroke patients. However, little has been reported that compares the clinical and physiological effects of a BCI intervention for upper limb stroke rehabilitation with those of conventional therapy. This study assesses the feasibility of an intervention with a BCI based on electroencephalography (EEG) coupled to a robotic hand orthosis for upper limb stroke rehabilitation and compares its outcomes to conventional therapy. Seven subacute and three chronic stroke patients (*M* = 59.9 ± 12.8) with severe upper limb impairment were recruited in a crossover feasibility study to receive 1 month of BCI therapy and 1 month of conventional therapy in random order. The outcome measures were comprised of: Fugl-Meyer Assessment of the Upper Extremity (FMA-UE), Action Research Arm Test (ARAT), motor evoked potentials elicited by transcranial magnetic stimulation (TMS), hand dynamometry, and EEG. Additionally, BCI performance and user experience were measured. All measurements were acquired before and after each intervention. FMA-UE and ARAT after BCI (23.1 ± 16; 8.4 ± 10) and after conventional therapy (21.9 ± 15; 8.7 ± 11) were significantly higher (*p* < 0.017) compared to baseline (17.5 ± 15; 4.3 ± 6) but were similar between therapies (*p* > 0.017). *Via* TMS, corticospinal tract integrity could be assessed in the affected hemisphere of three patients at baseline, in five after BCI, and four after conventional therapy. While no significant difference (*p* > 0.05) was found in patients’ affected hand strength, it was higher after the BCI therapy. EEG cortical activations were significantly higher over motor and non-motor regions after both therapies (*p* < 0.017). System performance increased across BCI sessions, from 54 (50, 70%) to 72% (56, 83%). Patients reported moderate mental workloads and excellent usability with the BCI. Outcome measurements implied that a BCI intervention using a robotic hand orthosis as feedback has the potential to elicit neuroplasticity-related mechanisms, similar to those observed during conventional therapy, even in a group of severely impaired stroke patients. Therefore, the proposed BCI system could be a suitable therapy option and will be further assessed in clinical trials.

## Introduction

It is estimated that worldwide, 24.9 million people are living with ischemic stroke sequelae, and there are approximately 11.6 million new cases per year, making stroke one of the leading causes of disability (Benjamin et al., 2018). Stroke sequelae include complete or partial paralysis of one hemibody, known as hemiparesis (Bruce, 2005). Treatment for hemiparesis focuses on motor rehabilitation strategies that aim to enhance neural plasticity, stroke’s primary recovery mechanism (Bruce, 2005; Pekna et al., 2012). These strategies are most effective during the acute and subacute phases of stroke (Lee et al., 2015; Branco et al., 2019). Specifically, upper limb motor recovery seems to occur predominantly during these stages (Borschmann and Hayward, 2020). However, it has been reported that motor rehabilitation of the upper limb is difficult to achieve for most patients; 6 months after stroke onset, approximately 65% of patients are unable to use the affected upper limb in their daily activities (Bruce, 2005; Lee et al., 2015). Therefore, assessing the efficacy of new upper limb rehabilitation technologies is currently of interest to research (Hatem et al., 2016; Bertani et al., 2017).

Brain-Computer Interfaces (BCI) is a promising technology for upper limb stroke rehabilitation. These systems allow users to control an external device by decoding their intentions from the central nervous system, typically from electroencephalogram (EEG) recordings (Wolpaw et al., 2002). BCI systems comprise four stages: brain signal acquisition, processing, external device control, and feedback. Mental rehearsal of movement, attempted movement, or motor intention (MI), elicits activations over the sensorimotor cortex (Monge-Pereira et al., 2017). Studies confirm that stroke patients can control a BCI using this MI. MI can elicit increased or decreased alpha (8–13 Hz) and beta (14–32 Hz) oscillations in the EEG with respect to baseline. These cortical activations, known as event-related desynchronization/synchronization (ERD/ERS) (Pfurtscheller and Lopes Da Silva, 1999), are similar to those produced by passive movement and motor execution (Carrillo-de-la-Peña et al., 2008; Kraeutner et al., 2014). There is some evidence that BCI based on MI and coupled to assistive robotic devices promotes neural plasticity by providing somatosensory feedback while the subject executes MI of the paralyzed upper limb (Remsik et al., 2016; Monge-Pereira et al., 2017). This closed-loop somatosensory stimulation has the potential to enhance motor-related cortical activity in healthy subjects (Cantillo-Negrete et al., 2019) and stroke patients (Ono et al., 2014), ultimately aiding in restoring function to the affected upper limb.

Although BCI systems with different feedback types, such as visual, robotic, and functional electrical stimulation (FES) devices, have shown great potential for upper-limb rehabilitation of stroke patients, their efficacy is under evaluation by different research groups. For a review, see López-Larraz et al. (2018) and Cervera et al. (2018). However, most of these studies are on chronic stroke patients (Ramos-Murguialday et al., 2013; Ang et al., 2014; Ono et al., 2014). Few studies of BCI coupled to robotic assistive devices have focused on patients in earlier stroke stages (Frolov et al., 2017). Moreover, to the authors’ knowledge, little has been reported that compares the effects of these devices for upper limb motor recovery after stroke to those of conventional therapy. Studies with the largest reported sample sizes have provided conventional therapy simultaneous to the intervention with the BCI coupled to a robotic device, without a direct comparison between them (Ramos-Murguialday et al., 2013; Frolov et al., 2017). Their direct comparison may help define a potential role for BCI interventions in the clinical environment for stroke patients. The implementation of these systems would reduce dependence on the availability of physiotherapists, which could effectively increase the amount of upper limb therapy stroke patients receive in the critical window after injury, thereby lessening the burden of stroke in healthcare systems worldwide.

Therefore, this study assesses the feasibility of an intervention with an MI-based BCI coupled with a robotic hand orthosis for upper limb stroke rehabilitation (referred to as ReHand-BCI) and compares patients’ outcomes with those obtained with conventional therapy. For this purpose, subacute and chronic stroke patients were recruited for a crossover pilot study, in which two groups of patients received both BCI and conventional upper limb therapy in a different sequence. The Fugl-Meyer Assessment of Upper Extremity (FMA-UE) and the Action Research Arm Test (ARAT) (Lang et al., 2006) were used for assessing upper limb motor recovery after each treatment. Hand dynamometry, transcranial magnetic stimulation (TMS), BCI performance, quantitative EEG, and user experience were also evaluated to complement the clinical measurements.

## Materials and Methods

### Patients

Patients meeting the following criteria were invited to participate by medical specialists from the stroke rehabilitation service of the medical institution in which this study was conducted. Inclusion criteria: adults (>18 years) diagnosed by a neurologist with ischemic stroke in either hemisphere confirmed by magnetic resonance imaging (MRI) or computed tomography (CT). No less than 2 months and no more than 12 months since the onset of stroke. Study subjects presented mild to severe hand paralysis [Motricity Index = 0, 11 or 19 (Demeurisse et al., 1980)] and were right-handed before the stroke. Subjects had normal or corrected to normal vision. Most had mild alterations in attention and memory processes according to the neuropsychological test NEUROPSI (Ostrosky et al., 2019) and demonstrated an adequate understanding of instructions according to the Boston Diagnostic Aphasia Examination (BDAE-3) (Goodglass et al., 2005). Exclusion criteria were: severe spasticity (Modified Ashworth Scale score > 2) in finger regions, severe aphasia (severity scale score ≥ 2), and history of other previous neurological lesions. Elimination criteria included the patients’ determination to withdraw from therapy, pain in the upper limb, epilepsy, seizures, or symptoms of any other neurological disorder during the study.

### ReHand-BCI System

The ReHand-BCI is controlled by MI of the stroke patients’ paralyzed hand and includes the following stages:

• Acquisition stage: EEG signals were recorded using a cap with 11 active electrodes (g.LadyBird, g.tec). Electrodes were placed on F3, C3, T3, P3, Fz, Cz, Pz, F4, C4, T4, and P4 according to the international 10-20 system. The reference electrode was placed in the right earlobe, and the ground electrode was placed in the AFz position. Each channel was amplified and digitalized with a g.USBamp amplifier from g.tec connected to a PC at a sampling rate of 256 Hz with 24-bit A/D resolution.
• Processing stage: Windows of one-second length were processed following these phases. The first phase consisted of the temporal filtering of the acquired multichannel EEG using subject-specific frequency bands, selected following an offline setup described below. A 30th-order FIR notch filter was also applied to the EEG signals. The second phase comprised spatial filtering using the Common Spatial Pattern (CSP) algorithm (Blankertz et al., 2008). Afterward, in the third phase, Linear Discriminant Analysis (LDA) was used for classification. Spatial filters and LDA coefficients were computed as described below. This processing stage is similar to the Filter-Bank Common Spatial Patterns (FBCSP) algorithm (Ang et al., 2008). The processing stage’s algorithms were programmed using the MATLAB 2015b software. A more detailed description of the online processing stage’s algorithms can be found in a previous work by Cantillo-Negrete et al. (Cantillo-Negrete et al., 2018).
• Robotic Hand Orthosis Control: If the processing stage detected MI of the stroke patients’ paralyzed hand, a Bluetooth wireless command was sent to a robotic hand orthosis fixed to that hand, which then provided passive flexion and extension of their paralyzed fingers (Martinez-Valdes et al., 2014). Patients were shown faces with different degrees of smiling expressions after a defined number of trials had elapsed (a run of the system). This feedback indicated the number of times the system correctly identified MI and activated the robotic hand orthosis.
• Computation of subject-specific parameters: These parameters were computed to set up the online BCI processing stage. First, acquired multichannel EEG were visually inspected to discard trials with excessive artifacts. Afterward, temporal filtering using six 30th-order FIR bandpass filters in the 8–12, 12–16, 16–20, 20–24, 24–28, and 28–32 Hz frequency bands was performed, and a 30th-order FIR notch filter was also applied to the EEG signals. Then, CSP spatial filtering was performed. Spatial filters were calculated by solving the eigenvalue of the covariance matrices of the MI and rest classes, which resulted in 11 spatial filters for each sub-band; thus, 66 features were computed for each trial. Next, the feature selection algorithm using Particle Swarm Optimization (PSO) (Shi and Eberhart, 1998) was applied to select the least number of features for which a higher classification accuracy could be obtained. PSO parameters were set based on previous studies (Cantillo-Negrete et al., 2017, 2018), with the stopping criteria of the algorithm set at either finding a combination of features for which 100% of classification accuracy could be reached or when 50 generations (iterations) had elapsed. Finally, the fourth phase is an LDA classifier, which uses a subset of the 66 features obtained with CSP as input. This combination of temporal and spatial filtering and particle swarm optimization used for feature selection is referred to as FBCSP+PSO. After this process, subject-specific frequency bands, CSP filters, and LDA coefficients were used for the online implementation of the algorithm.

**FIGURE 1:**
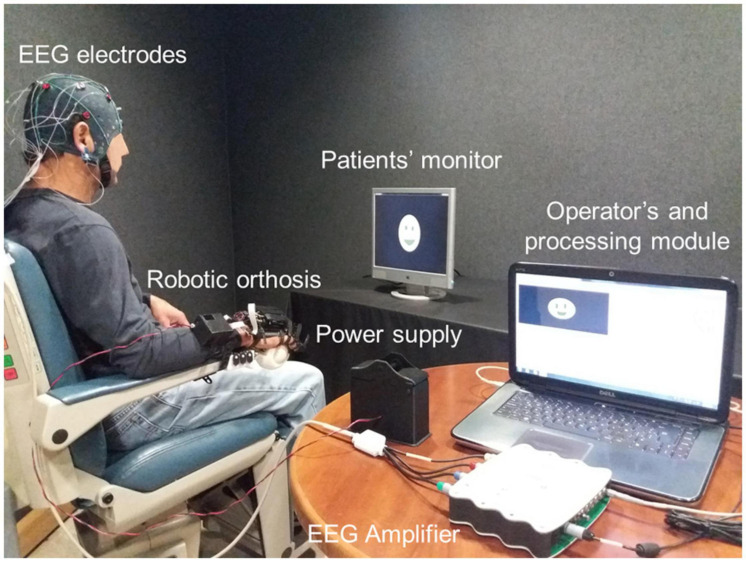
ReHand-BCI system. Acquisition stage comprised of EEG electrodes, amplifier, and A/D converter. The processing module included CSP, PSO, and LDA programmed in a PC, which also has a graphical user interface for setting up therapy parameters. Commands are sent through wireless communication from the PC to the robotic hand orthosis.

Previous studies had shown that stroke patients could control the ReHand-BCI with the 11 electrode configuration (Cantillo-Negrete et al., 2017, 2018) and that the ReHand-BCI could be set up in less than 10 min. To determine if the robotic hand orthosis was apt for BCI feedback, its capability to promote cortical activations above the sensorimotor cortex was first confirmed in healthy subjects (Cantillo-Negrete et al., 2019). The ReHand-BCI system is depicted in Figure 1.

### Study Design

**FIGURE 2:**
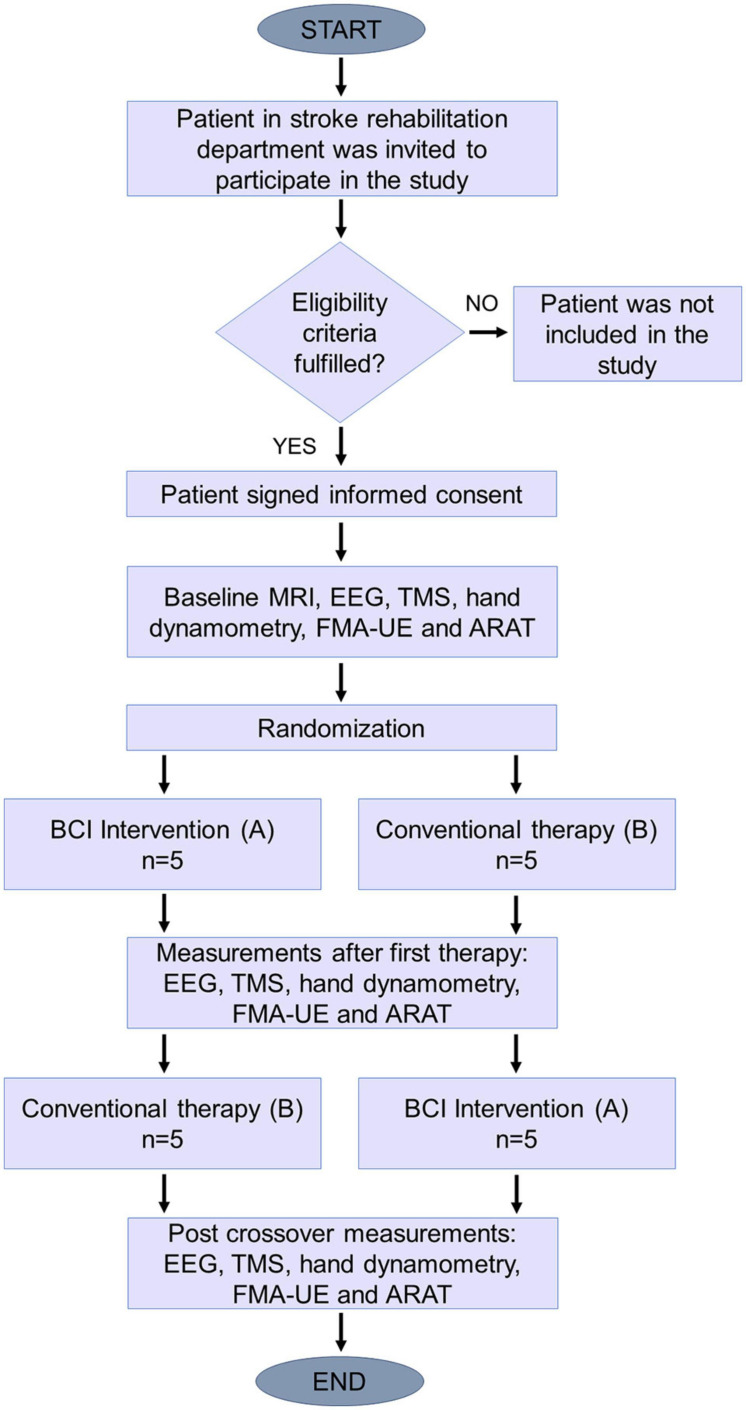
Study design diagram. After eligibility criteria were assessed, patients were assigned randomly to one of the two sequences.

A randomized crossover pilot study was planned with a convenience sample of at most 10 subacute and chronic stroke patients, as is recommended in feasibility studies for early clinical evaluation of medical devices (FDA, 2011; Billingham et al., 2013). Patients were randomly allocated to one of two different sequences of therapeutic intervention. The sequence for group 1 (AB) was comprised of 12 sessions of BCI therapy (A) followed by 12 sessions of conventional upper limb therapy (B). While the sequence for group 2 was, first 12 sessions of conventional therapy (B), then 12 sessions of BCI therapy (A). Each therapeutic intervention consisted of three sessions per week for 4 weeks. Standard treatment for stroke sequelae, including lower-limb and speech therapy, was unrestricted for the study subjects. The experimental sessions were added to the patients’ standard treatment, with both groups receiving conventional upper limb therapy. Simple randomization was programmed in Microsoft Excel^®^ and was blinded for all study participants, except for the researcher who executed the program. Figure 2 shows a diagram of the experimental study’s design.

Before the group assignment, baseline clinical assessments were obtained for each patient, including FMA-UE and ARAT. Stroke diagnosis was confirmed with an MRI study. Physiological measurements such as hand dynamometry (HD), TMS and EEG, were also assessed at baseline. After each group’s first therapy, FMA-UE, ARAT, HD, TMS, and EEG were measured again. Then after the crossover therapy, these same measurements were repeated.

### Baseline EEG

**FIGURE 3:**
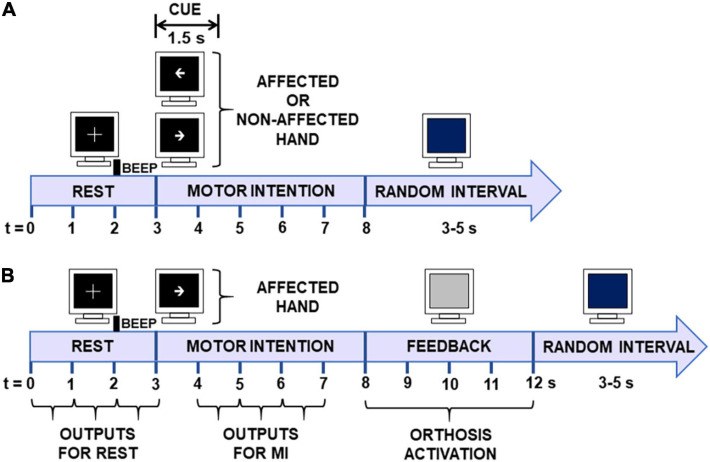
Time diagrams of offline and online trials. **(A)** Baseline EEG recordings. During the first 3 s, patients rested with their eyes open (REST). From 3 to 4.5 s, an arrow signaled them to initiate MI with the affected or unaffected limb. MI was performed from 3 to 8 s. The data from REST (0–3 s) and MI (4–7 s) periods were used for posterior EEG analyses. **(B)** Online BCI trials. The first 3 s was used for classification of the REST condition, and from 4 to 7 s for MI of the affected limb.

Baseline EEG was recorded to obtain the patients’ offline performance and rule out exclusion criteria such as other neurological pathologies. EEG acquisition was performed in two sessions to avoid patient fatigue. In each session, patients performed four tasks; affected hand motor imagery, non-affected hand motor imagery, affected hand movement intention, and non-affected hand movement execution (80 trials per task, 320 trials in total). Only the 80 trials of affected hand motor intention were used to compare ERD/ERS at baseline and after therapies. The post-therapy EEGs were acquired in a single session of 60 trials similar to a therapy session. Recordings were done in a sound-attenuated closed room, with a computer screen placed at approximately 1.5 m from patients seated in a comfortable armchair. The electrode positions and recording system were the same as with the ReHand-BCI (see section “ReHand-BCI System”). Visual and auditory cues, based on the Graz paradigm (Pfurtscheller and Neuper, 2001), were shown to patients, instructing them when to perform MI. Figure 3A shows the timing of the visual and auditory cues during baseline recordings.

### BCI Therapy

Sessions were conducted in the same sound-attenuated room and under the same experimental conditions as those used for baseline EEG (see section “Baseline EEG”). First, an EEG cap with 11 electrodes as mentioned above was adjusted to the patient’s head; next, the robotic hand orthosis was fitted to the stroke patients’ affected hand. Afterward, visual and auditory cues signaled patients when to initiate MI of their hand, which involved the intention of continuous finger flexion and extension. Each session included three runs of 20 trials each (60 trials, i.e., attempts of MI per session). The time structure of each trial was as follows. The first 3 s were of REST, then an arrow pointing at the stroke patient’s affected hand signaled the onset of MI. The arrow was shown for 1.5 s, after which the screen turned black until the 8 s mark of the trial. From 8 to 12 s, feedback was either provided by the robotic hand orthosis, which passively flexed then extended the fingers, or by non-activation of the orthosis. During the feedback period, the screen turned gray. From 12 s onward, a blue screen signaled to patients that they could move or relax and lasted for a random interval of 3–5 s to prevent habituation. Following this interval, a new trial started, or if 20 trials had already elapsed, then a resting period of a least 1 min was observed before the next run started. The time structure of the trials is shown in Figure 3B. The duration of each session ranged from 30 to 40 min, depending on the time needed to place the electrodes and fit the robotic orthosis.

In the first session, the robotic orthosis was activated in all 60 of the trials to obtain MI and REST data used to set the BCI processing stage of the following session and for patients to get familiarized with the system. From the second session onward, the BCI processing stage was configured (see section “ReHand-BCI System”) with data from the previous session, and the robotic orthosis was only activated if at least two 1-s time windows of the MI interval were correctly classified. Patients’ online performance was computed for each of the 12 sessions. For each trial, classification accuracy was computed as the percentage of correct classified 1-s windows of REST and MI of each trial (see Figure 3B). Then, the classification accuracy of all 60 trials recorded per BCI intervention session was averaged for each patient. Afterward, the grand averaged classification accuracies were computed.

At the end of each patient’s last session of BCI, they answered the NASA Task Load Index (NASA-TLX) (Hart and Staveland, 1988) and System Usability Scale (SUS) (Brooke, 1996) questionnaires that assessed the subjective mental workload and the quality of the user experience when interacting with the ReHand-BCI. The raw NASA-TLX was used (the version that does not incorporate a multi-dimensional rating procedure), as evidence suggests that this version might increase experimental validity (Bustamante and Spain, 2012; Hart, 2012).

### Conventional Therapy

**FIGURE 4:**
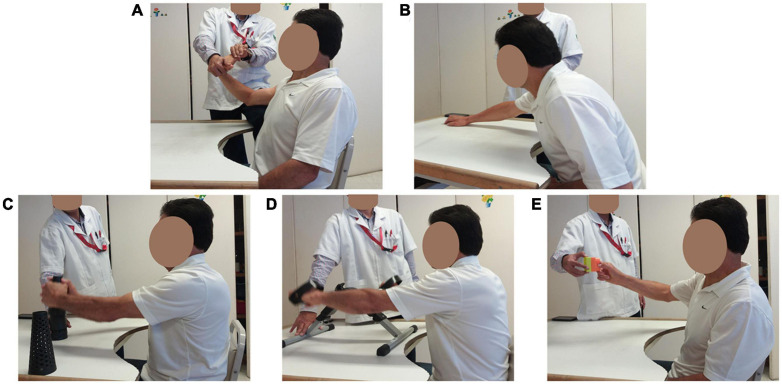
Conventional therapy. **(A)** Finger and wrist stretching exercises. **(B)** Arm stretches. **(C)** Gross pinch and lifting objects. **(D)** Gross pinch and rotating arm exercise. **(E)** Fine pinch grip lifts.

Conventional therapy was conducted in identical environmental conditions and by the same experienced professional therapist from the brain plasticity service. The intervention consisted of activities aimed at improving function of the affected upper limb, including neurofacilitation techniques for strengthening muscle tone and increasing voluntary movement, sensitivity reeducation, stretching, activities for the improvement of fine and gross grip, mobility arcs, muscle strength, and coordination. The duration of each session ranged from 30 to 40 min, depending on the time it took patients to complete the same exercise routine. Figure 4 shows a patient’s execution of upper-limb rehabilitation exercises during conventional therapy.

### Outcome Measurements

Clinical guidelines state that the FMA-UE should be used for sensitivity and motor evaluations of stroke patients and the ARAT for motor assessment of upper-limb function (Winstein et al., 2016). FMA-UE is the primary outcome measure reported by studies aiming to assess BCI efficiency for stroke rehabilitation (Ramos-Murguialday et al., 2013; Ang et al., 2014, 2015; Frolov et al., 2017). The ARAT has also been used as a primary or complementary outcome measure in BCI-based stroke rehabilitation studies (Frolov et al., 2017). Therefore, these two clinical scales were used to assess patient’s upper-limb motor recovery. The FMA-UE is a 0–66 item scale, in which a higher score represents less upper-limb motor impairment. The ARAT score ranges from 0–57; 0 is given if no upper-limb movements could be executed. FMA-UE and ARAT measurements were taken three times for each patient, at baseline, after the first therapy, and after the crossover therapy.

For HD, grip and pinch strength of the affected hand were assessed using the Biometrics E-link evaluation system. The dynamometer was placed in the second handle position. First, three measurements of grip strength were taken of the unaffected hand; if the coefficient of variation (Kroemer and Marras, 1980) was above 15%, then the measurements were repeated. Afterward, the affected hand was assessed using the same procedure. Patients had a 1-min rest period between measurements. Pinch strength was assessed using the same procedure but by measuring the force exerted between the index and thumb fingers. These measurements were used to compute the average grip and pinch strength for each hand per session.

For TMS, a baseline MRI was used for the neuronavigation system. It was obtained by acquiring an image sequence using a 64-channel head coil (Siemens Skyra 3.0T, Erlangen, Germany). The anatomical scan collected 192 mm × 1.2 mm thick slices with a voxel size of 1 mm × 1 mm × 1.2 mm, repetition time and echo time (TR/TE) of 2050/2.43 ms, field of view (FOV) 256 × 256 and matrix 256 × 256, and a coronal sequence for neuronavigation.

Single-pulse TMS motor evoked potentials (MEPs) were recorded by a physician trained to use a Rapid2 MAGSTIM device with a figure-of-eight coil. A bipolar EMG with a sampling frequency of 1,500 Hz was placed in the first dorsal interosseous muscle of both affected and unaffected hands. Each TMS session started with an initial mapping of the sensorimotor cortex of the patients’ unaffected hemisphere (UH), using MRI-guided stereotaxic neuronavigation, following the relative-frequency method described by Rossini et al. (Rossini et al., 2015) to determine the resting motor threshold (RMT). Then, stimulus intensity was lowered in steps of 1% MSO until MEPs were not detected in less than five out of 10 trials. Next, using this stimulus intensity plus 1 (i.e., the RMT), 30 trials were recorded to compute the MEP amplitude of the UH motor cortex. Afterward, the same procedure was used for the affected hemisphere (AH). It has been reported that at least 20 trials of MEP recordings should be made for reliable assessment of single-pulse MEPs (Biabani et al., 2018). Therefore, each session’s MEP peak to peak amplitude was computed using an automatic recognition software that averaged maximum and minimum values from the 20 trials with the fewest artifacts (Tecuapetla-Trejo et al., 2020). MEP amplitude was used to assess the corticospinal tract integrity of patients before and after the interventions.

EEG recordings were first filtered with a 30th-order FIR bandpass filter from 8 to 32 Hz and a 30th-order FIR notch filter from 58 to 62 Hz. Afterward, a common average reference (CAR) spatial filter was applied on each channel to reduce reference placement effects on the EEG signal (Bertrand et al., 1985). Visual inspection of the EEG readouts was performed to identify and reject trials with artifacts. Alpha (8–13 Hz) and beta (14–32 Hz) ERD/ERS (Pfurtscheller and Lopes Da Silva, 1999) were computed for each trial and channel, using Equation 1.

(1) $$\%ERD/ERS=\frac{P_{M\,I-}\,P_{r\,e\,s\,t}}{P_{r\,e\,s\,t}}*100$$

Where *P*_*MI*_ is the MI task’s power, and *P*_*rest*_ is the averaged power during the rest condition. Power was computed using the complex Morlet wavelet transform, as reported by Tallon-Baudry et al. (1999).

For the posterior statistical analysis, 3-s windows, including REST (0–3 s) and MI (4–7 s) intervals were extracted. Grand average topographic maps were computed with the ERD/ERS from baseline, after BCI, and after conventional therapy. To unify the data collected from patients’ affected and unaffected hemispheres, ERD/ERS for the left hemisphere’s channels (F3, C3, T3, and P3) of patients with right hemisphere lesions were interchanged with right hemisphere channels (F4, C4, T4, and P4). This change allowed all patient’s cortical activity of the affected hemisphere to be shown over the left hemisphere’s channels (FAH, CAH, TAH, and PAH) and the unaffected hemisphere’s activity to be shown over the right channels (FUH, CUH, TUH, and PUH).

### Statistical Analysis

Outcome measures obtained at baseline, after BCI, and after conventional therapy were assessed for Gaussian distribution with a Lilliefors-corrected Kolmogorov-Smirnov test. Differences between groups’ baseline clinical measurements were evaluated using non-paired *t*-tests (Wilcoxon rank-sum tests for non-Gaussian distributions). Repeated measurements analysis of variance (ANOVA) was used if data followed a Gaussian distribution; if not, an exact Friedman test was used to assess if significant differences were found between baseline and after both therapies. Multiple comparisons testing with paired *t*-tests (Wilcoxon signed-rank tests for non-Gaussian distributions) was performed with a Bonferroni correction applied. The binary outcome of the TMS results (presence or not of resting motor threshold) was analyzed with the McNemar test to assess differences between the two therapies, and a Cochran’s Q test was used to identify differences between repeated measurements. To assess the reliability of the BCI, the practical level of chance, defined by Müller-putz et al. (2008) as the upper confidence interval of a random classifier’s accuracy, was computed with a binomial distribution using a significance level of 0.05. For all statistical tests, the significance level (α) was set at 0.05. Statistical analysis was done using the SPSS v.17 software.

## Results

### Clinical Outcomes

In total, 11 patients were included in this study; patient P2 was eliminated after presenting a mild convulsive episode after the conventional therapy (B) and before the BCI intervention (A). The patient (male, 53 years old, 160 days since stroke onset) did not report previous seizure events at enrollment in the study nor during the baseline TMS session (MEPs were elicited in both hemispheres). His elimination from the study was due to the possibility that another neuropathology triggered the seizure event and could affect his motor recovery. Demographic data for each patient is shown in Table 1.

**TABLE 1:** Demographic data of stroke patients included in the present study, including therapy sequence allocation.

**ID (Sequence)**	**Gender**	**Hemiparesis**	**TSSO (days)**	**Age (years)**	**Lesion, type, and location**	**Baseline FMA-UE, ARAT**	**Baseline grip, pinch strength (Kgf)**	**MEPs in AH**
P1(AB)	Female	Right	280	54	Subcortical. L. Lentiform Nucleus, L. Internal Capsule, and L. Thalamus.	12, 0	0.6, 0.1	No
P3(BA)	Female	Left	81	85	Subcortical. R. Pontine Tegmentum.	13, 3	1.3, 0.7	Yes
P4(AB)	Female	Right	218	58	Subcortical. L. Lentiform Nucleus, L. Internal Capsule.	9, 5	0.5, 0	Yes
P5(BA)	Female	Left	146	54	Cortical-Subcortical. R. Insula, R. Lentiform Nucleus, R. Internal Capsule.	9, 0	0.5, 0	No
P6(BA)	Male	Left	37	43	Subcortical. R. Pontine Tegmentum.	8, 0	0.2, 0.2	No
P7(AB)	Male	Right	100	48	Subcortical. L. Internal Capsule.	15, 0	1.9,0	No
P8(BA)	Male	Right	97	53	Cortical. L. Insula	14, 3	0.7, 0	No
P9(AB)	Male	Right	260	63	Subcortical. L. Lentiform Nucleus, L. Internal Capsule.	59, 21	9.7, 2.3	Yes
P10(BA)	Male	Left	87	65	Subcortical. R. Internal Capsule. R. Thalamus.	12, 3	0, 0	No
P11(AB)	Female	Left	98	76	Subcortical. R. Internal Capsule.	24, 8	0.8, 0.5	No
**Mean (SD)**			**140 (83)**	**59.9 (12.8)**		**17.5(15.3), 4.3(6.4)**	**1.62(2.9), 0.38(0.72)**	

*TSSO, Time since stroke onset; AH, Affected Hemisphere.Patients’ age and time since stroke onset are relative to the beginning of the study. The presence of Motor Evoked Potentials (MEPs) was assessed with Transcranial Magnetic Stimulation before interventions. The bold values are the mean and the standard deviation of values of the fourth and fifth columns of the table.*

The recruited sample was balanced regarding gender and affected hemisphere. Seven patients were in the subacute (7 days to 6 months) and three in the chronic (>6 months) phase of stroke (Bernhardt et al., 2017). Their age range was between 43 and 85 years at the beginning of the study. Most patients presented subcortical stroke (*n* = 8), one patient had a cortical stroke (*n* = 1), and one patient had a cortical-subcortical stroke (*n* = 1). The patients’ age was similar for sequence AB and BA, *t*(8) = −0.023, *p* = 0.398. However, there was a significant difference in time after stroke onset, *t*(8) = 2.38, *p* = 0.023. The baseline clinical and HD data were similar for both sequences (FMA-UE, *p* = 0.214; ARAT, *p* = 0.326; grip strength, *p* = 0.167; pinch strength, *p* = 0.683). Table 2 shows FMA-UE, ARAT, and HD scores at baseline and after both therapies.

**TABLE 2:** Clinical and hand dynamometry (kgf) outcomes.

**Outcome Measure**	**Baseline (BL)**		**BCI (A)**		**Conventional (B)**		***p***	**Pairwise Comparison**
	**Mean (SD)**	**Median [IQR]**	**Mean (SD)**	**Median [IQR]**	**Mean (SD)**	**Median [IQR]**		
FMA-UE	17.5 (15.3)	12.5 [9;17]	23.1 (16.1)	**15.5** [13;30]	21.9 (15.5)	15 [12;35]	0.014	BL > A*; BL > B*; A = B
ARAT	4.3 (6.4)	3 [0;6]	8.4 (10.1)	**4.5** [0;16]	8.7 (11.3)	**4.5** [0;16]	0.001	BL > A*; BL > B*; A = B
HD(grip)	1.62 (2.9)	0.65 [0.4;1.5]	2.42 (3.3)	**1.35** [0.3;1.4]	1.58 (2.6)	0.90 [0.7;3.2]	0.682	–
HD(pinch)	0.38 (0.72)	0.05 [0;0.6]	0.45 (0.48)	**0.30** [0.8;0.3]	0.29 (0.48)	0.15 [0.1;0.7]	0.376	–

HD, Hand Dynamometry; SD, Standard Deviation; IQR, Interquartile Range; *p < .017.Highest median values are shown in bold.

An exact Friedman test showed a statistically significant difference between FMA-UE scores measured at baseline, after BCI therapy, and after conventional therapy (Table 2). *Post hoc* tests using an exact Wilcoxon signed-rank test with a Bonferroni-adjusted alfa level showed a statistically significant increase in FMA-UE scores after BCI and conventional therapy compared to baseline. However, the scores after BCI and conventional therapy were not significantly different. FMA-UE scores measured before and after BCI therapy indicated an average gain of 2.4 (SD = 3.2), and before and after conventional therapy showed an average gain of 3 (SD = 7.7). Therefore, after completing the interventions, the mean FMA-UE score increased by 5.4 points.

Similarly, an exact Friedman test showed a statistically significant difference in ARAT scores measured at baseline, after BCI therapy, and after conventional therapy (Table 2). *Post hoc* tests demonstrated that ARAT scores after BCI and after conventional therapy were significantly higher than baseline. However, BCI and conventional therapy scores were not significantly different. ARAT scores measured before and after BCI therapy indicated an average gain of 2.1 (SD = 3.2), and before and after conventional therapy showed an average gain of 3 (SD = 5.1). So, after completing the interventions, the mean ARAT score increased by 5.1 points. An average FMA-UE gain of 4.6 (SD = 7.5) was observed in patients after the first intervention, while an average FMA-UE gain of 0.8 (SD = 2.5) was observed after the second intervention. An average ARAT gain of 3.4 (SD = 5.1) was observed in patients after the first intervention, followed by a gain of 1.7 (SD = 2.9) after the second intervention.

### Hand Dynamometry

The outcomes for grip and pinch strength of each stroke patients’ paralyzed hand are presented in Table 2. Although an exact Friedman test shows no statistical difference in grip strength measured at baseline, after BCI, and after conventional therapy, median grip strength of the patients’ paralyzed hand was higher after BCI. Similarly, there was no statistically significant difference between pinch strength measured at baseline and after both interventions; however, median pinch strength after BCI was higher than after conventional therapy.

### TMS Outcomes

**FIGURE 5:**
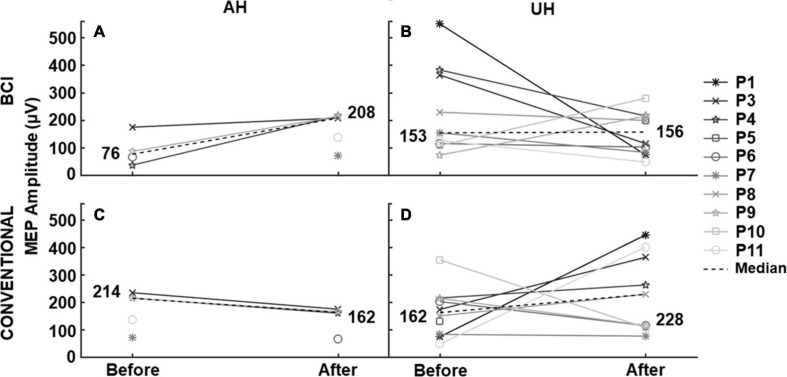
MEP amplitude before and after each intervention. TMS elicited median MEP amplitude in patients’ affected (AH) and unaffected (UH) hemispheres, before and after the BCI and conventional therapies. Patients that did not present MEPs are not shown in the measurements.

Figure 5 shows the MEP amplitudes of each patient before and after each therapy. Patients presented the most pronounced differences in the AH. The highest difference in median MEP amplitude was observed in the AH after the BCI intervention (Δ = 132 μV), while after conventional therapy, this difference was less pronounced (Δ = −52 μV). In the UH, differences of median MEP amplitude were not as pronounced as in the AH (Δ = 3 μV after the BCI and Δ = 66 μV after conventional therapy). Two patients that did not present MEPs in their AH before the BCI presented them after the intervention, whereas one patient that did not present MEPs in the AH before conventional therapy presented them after the intervention.

Median resting motor thresholds in the UH were between 63–66% of the maximum output of the TMS system, while in the AH, they were between 87–100%. As resting motor thresholds and MEPS were not detected in all patients, statistical analyses could not be done on TMS continuous measurements. Therefore, MEPS in the affected hemisphere were analyzed as binary outcomes, considered either detected or not. A McNemar’s test determined no statistical difference (*p* > 0.05) between BCI and conventional therapy regarding MEP presence. Besides, a Cochran’s test showed that statistically, there was no difference in detection/presence of MEPS between baseline, after BCI, and after conventional therapy (*p* > 0.05).

### Quantitative EEG

**FIGURE 6:**
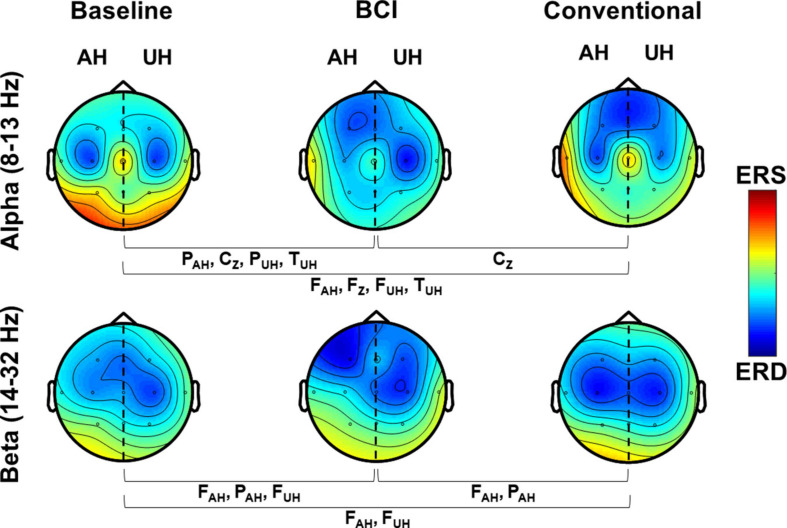
Grand average ERD/ERS maps in alpha and beta. ERD/ERS is shown over affected and unaffected hemispheres during MI (4–7 s). Channels that presented significant differences (*p* < 0.017) between measurements are shown.

Figure 6 shows grand averaged ERD/ERS during MI of patients’ affected hand at baseline and after interventions. Also, for each band, the results of exact Friedman tests, with Bonferroni correction for comparing channels’ ERD/ERS are given. In alpha (8–13 Hz), comparisons with baseline activations showed that after the BCI intervention, patients presented significant ERD/ERS differences in more regions (central, temporal and parietal) than the regions with significant differences observed after conventional therapy (frontal and temporal). The only significant difference noted between BCI and conventional therapies was in the central sagittal area, with more pronounced ERD elicited after the BCI therapy. In beta (14–32 Hz), ERD/ERS differences after the BCI compared to baseline were detected in frontal and parietal regions. After the conventional therapy, significant differences were evident in frontal channels compared to baseline measurements. Differences between ERD/ERS after the BCI and conventional therapies were only significant in the frontal and parietal regions of the AH.

### BCI Performance

**FIGURE 7:**
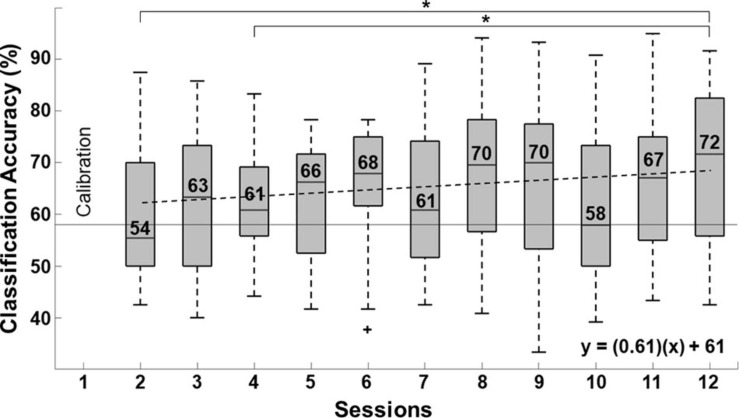
Linear trend of BCI performance measured with the percentage of classification accuracy (%CA). Significant differences (*p* < 0.005) in %CA between consecutive sessions are indicated with (*). Outliers are also marked (+). The practical level of chance is shown as a horizontal broken line.

The grand average percentage of classification accuracy (%CA) during each of the 12 BCI sessions is shown in Figure 7. Since the last session’s (session 12) percentage of classification accuracy (%CA) had the highest median, we only compared this session’s %CA with the %CA of the other sessions (session 2 to 11). Session 1 was not compared because it was a calibration session. Therefore, *post hoc* analysis with Wilcoxon signed-rank tests was done with a Bonferroni correction (*p* = 0.05/10) since 10 comparisons were performed. Significant differences were only observed between session 12 with sessions 2 and 4. Only the second session’s median %CA was below the practical level of chance (58%). A positive linear trend of 0.61 was computed from %CA and session number using linear regression analysis (R^2^ = 0.47, *p* = 0.002), which suggests that classification accuracy increased across sessions.

### User Experience With the BCI

**FIGURE 8:**
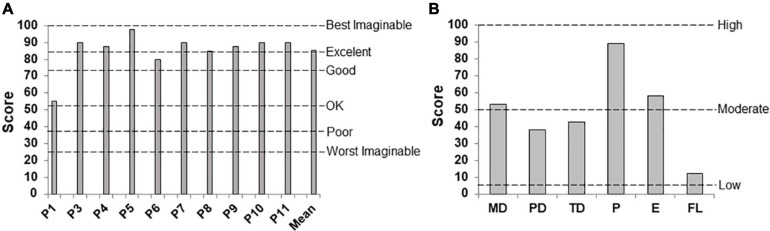
User experience after concluding the BCI intervention. **(A)** System Usability Scale and **(B)** NASA-TLX, showing averaged scores assigned to the factors of Mental Demand (MD), Physical Demand (PD), Temporal Demand (TD), Overall Performance (P), Effort (E), and Frustration Level (FL).

Figure 8A shows the average SUS scores given by each patient that underwent BCI therapy. For the SUS graph, patients rated different aspects of the system’s usability with a descriptive adjective ranging from “best imaginable” to “worst imaginable,” per the study of Bangor et al. (2009). Eight patient’s assessment of the system was within the best possible range. One patient rated the system in the second-best range (P6), and one patient graded its usability in the acceptable range (P1). Figure 8B displays averaged NASA-TLX scores. Adjectives words were added to figure based on the rating scale’s endpoints. Their responses indicated high overall performance and low frustration with the system. Also, patients stated that using the system required moderate mental demand and effort and low to moderate physical and temporal demand.

## Discussion

While FMA-UE and ARAT measurements showed no significant differences in patients’ upper limb motor recovery between the BCI and conventional therapy, patients were less impaired after either intervention, which suggests both interventions effectively increased upper limb motor function in stroke patients. A relevant point to consider is that the conventional therapy involved gross pinch and different upper-limb movements, unlike the BCI, that only comprised gross pinch. However, clinical outcomes showed that the effects of both interventions were similar. Therefore, although the BCI therapy only targeted finger flexion-extension, it was hypothesized that the ReHand-BCI could also benefit motor recovery due to the closed-loop communication between the patient and their affected upper limb that it provides. This hypothesis is reinforced by other BCI studies that reported lower stroke rehabilitation outcomes of control groups that only received passive movement feedback (Li et al., 2014) or sham feedback (Ramos-Murguialday et al., 2013; Frolov et al., 2017).

In the present study, changes in FMA-UE after BCI were between 2.4 ± 3.2, which is within the range of the study of Ang et al. (Ang et al., 2015) (4.55 ± 6.1) and Ramos-Murguialday et al. (2013) (3.4 ± 2.2). It is important to remark that the mean gain of FMA-UE scores in our study was lower than those reported in the aforementioned studies. However, in the study of Ang et al., mean FMA-UE scores at baseline were 26.3 ± 10.3, while in the present study, they were 17.5 ± 15.3. This difference suggests that our patients had more upper limb motor impairment at baseline, and therefore, as stated in other works, their probability of achieving upper limb motor recovery was lower (Bruce, 2005; Lee et al., 2015). Also, Frolov et al. (2017) reported a median FMA-UE difference of three points in subacute stroke patients after therapy with a BCI, which is within the observed range of 2.4 ± 3.2 in our study. However, these FMA-UE differences were below the range of 7.2 ± 2.3 reported in another study of Ang et al. (2014) after 1.5-months of BCI coupled to a robotic hand knob. Possible explanations for these results could be the longer duration of their BCI therapy and lower baseline upper limb motor impairment (33 ± 16.2). Therefore, the upper limb motor recovery of the patients in this study was similar to what has been reported for other BCI coupled to robotic assistive devices with a comparable duration of therapy and upper limb impairment at baseline, and lower than studies reporting longer BCI interventions, or patients with lower baseline upper limb motor impairments.

To the authors’ knowledge, only Frolov et al. (Frolov et al., 2017) have reported stroke patients’ ARAT scores after upper limb rehabilitation using a BCI system coupled to a robotic assistive device. In their work, a median difference of two points in the ARAT score was reported after the BCI therapy in subacute stroke patients, which is within the range observed in the present study. This finding implies that the ReHand-BCI could elicit a similar degree of motor recovery as a state-of-the-art BCI system coupled to a robotic assistive device designed for stroke patients’ upper limb neurorehabilitation.

After each therapy, mean differences in the FMA-UE were not higher than the minimal clinically important difference (MCID) of 5.25 (Page et al., 2012), nor was the ARAT mean difference for any individual intervention higher than the MCID for ARAT of 5.7 (Van Der Lee et al., 2001). However, the mean gain in the FMA-UE observed in the present study after finalizing the interventions (*M* = 5.4) was higher than the MCID. Also, the MCID of the ARAT is close to the mean gain observed after completing the interventions in this study (*M* = 5.1); therefore, even to a small degree, patients’ recovery may be noticeable in their daily living activities. It also highlights that for stroke patients with severe baseline impairments to achieve clinically relevant gains in upper limb recovery, the duration of treatment should be more than 1-month and include more than 12 sessions. Also worthy of mention is that patients showed a higher average recovery in both FMA-UE and ARAT after the first therapy, compared to the recovery observed after the second therapy. Greater recovery after the first period of intervention, compared to the second, was also reported by Ang et al. (2014) since an average gain of 5.8 points in the FMA-UE score during three intervention weeks was followed by a lower gain of 1.4 after another 3 weeks of intervention in stroke patients. The lower motor recovery gain observed during the second intervention could have been caused by attenuation of treatment-driven functional recovery mechanisms due to less availability of residual neuronal substrate used for compensatory cortical reorganization processes (Stern, 2009; Alawieh et al., 2018). However, the effects of BCI and conventional treatments over time need to be assessed with a larger sample.

Most patients included in the present study were in the subacute stage of stroke, so spontaneous recovery was likely a contributing factor to patients’ observed clinical outcomes. However, the magnitude of spontaneous recovery has been reported to be correlated with initial stroke severity (Cassidy and Cramer, 2017; Delavaran et al., 2017), and subacute patients with low scores of FMA-UE have been reported to have a lower degree of spontaneous recovery (Franck et al., 2017). Only one of the seven subacute patients that participated in the present study had moderate impairment (a score of 23 in FMA-UE), while the others had severe impairment (0–22 in FMA-UE) (Woodbury et al., 2013). Furthermore, most subacute patients had severely compromised corticospinal tract integrity, as shown by an absence of MEPs in their AH before interventions; which has been related to lower recovery prognosis (Stinear et al., 2017). Therefore, the recovery observed in the subacute patients of our study was likely to be associated with the effects of the BCI and conventional therapies.

Grasp and pinch dynamometry showed no significant differences between measurements taken at baseline or after either therapy. However, the highest median scores for both grasp and pinch were observed after the BCI. Also, after the BCI intervention, relative changes in median grasp strength were 108% higher than baseline measurements and 20% higher than a study that used an MI-based BCI without an assistive robotic device, reported by Prasad et al. (Prasad et al., 2010). The gain in grip strength after 1 month of the BCI intervention (Mdn = 0.65 kg) amounted to almost half the grip strength gain reported in a sample of severely impaired stroke patients (Mdn = 2 kg) after 12 months of conventional therapy (Franck et al., 2017). These results suggest that the ReHand-BCI may increase grip strength in patients’ affected hands.

The majority of patients did not present MEPs when TMS was applied over their AH, which reinforces the statement that most had severe upper limb motor impairment and suggests that the integrity of the AH corticospinal tract of most patients was compromised. It also indicates that most patients in the present study had a poor prognosis. The PREP algorithm proposed by Stinear et al. (Stinear et al., 2017) predicts the potential for upper limb motor recovery after stroke; it shows an association between the absence of MEPs in the AH and adverse prognosis. Interestingly, two patients who did not present MEPs in the AH at baseline presented them after the BCI intervention. Furthermore, higher median MEP amplitudes over the AH after the BCI intervention could imply that neuroplasticity effects led to an increase in these patients’ corticospinal integrity. Integrity of the corticospinal tract in the AH, measured with TMS, has been associated with decreased upper limb motor impairment in stroke patients (Delvaux et al., 2003; Stinear et al., 2017). However, due to the low number of patients that presented MEPs in the AH and the difficulty of finding MEPs in the AH of stroke patients overall, these observations must be confirmed in a larger sample.

After the BCI therapy, quantitative EEG showed that in alpha band, ERD was significantly more pronounced in central sagittal regions than at baseline and after conventional therapy. This finding implies that when patients’ performed MI of their affected hand during BCI therapy, more areas of the sensorimotor cortex presented enhanced motor-related activity than at baseline or after conventional therapy. A possible explanation for this could be that the passive movement feedback provided by the robotic orthosis reinforced closed-loop communication between the sensorimotor cortex and the affected upper limb. As reported previously by our group (Cantillo-Negrete et al., 2019) and others (Ang et al., 2015), in healthy individuals and stroke patients, respectively, subjects presented more activity in alpha and/or beta after training with the BCI compared to baseline in areas not usually associated with motor tasks, such as frontal, parietal, and temporal regions. Also, frontal regions showed higher activations in beta after the BCI compared to conventional therapy, which could be due to an enlargement of the motor cortex area during motor-related tasks. This compensatory mechanism was observed by Ward et al. (2003) in severely impaired stroke patients using fMRI, in a review by Cassidy and Cramer (Cassidy and Cramer, 2017), and was hypothesized after the preliminary results of a longitudinal analysis of ERD/ERS across therapy sessions (Carino-Escobar et al., 2019). Although some BCI aimed for stroke rehabilitation only use activations from the AH (López-Larraz et al., 2018), the ReHand-BCI (that uses data from both hemispheres) could be enhancing cortical activations usually present in motor-related regions of the cortex and other areas. This enhancement may be associated with the neural plasticity observed during motor-related tasks in patients with severe motor impairment.

Despite variability in patients’ BCI system control, a trend toward higher %CA was observed across therapy sessions. Patients’ median control was only below the practical level of chance in the second session (which was the first session patients attempted to control the BCI) and was highest in the last session (72%). Increased %CA across sessions with a BCI coupled to robotic assistive devices has been previously reported in stroke patients by Ramos-Murguialday et al. (2013) and Frolov et al. (2017). In the present study, the only significant differences in patients’ BCI control were observed between sessions 2 and 4 and the session with the highest median (session 12). This indicates that patients were achieving higher accuracy scores from the fifth session onward, which is slightly sooner than patients in the study by Ramos-Murguialday et al. (2013), whose degree of control was higher from the seventh session forward. In the 12th session of the present study, patients obtained a median maximum accuracy with the ReHand-BCI of 72% [55.8, 82.5%]. These degrees of control are in the same range as those reported by Ang et al. (2011) of an average of 74% in 46 stroke patients, using a 27-electrode system. However, in the last session with the ReHand-BCI, patients’ median accuracy was lower than the maximum accuracy range of 97.5–96.2% reported by Irimia et al. (2018) in five stroke patients controlling a BCI using 64 electrode positions, but within their minimum reported accuracy range (82.5–60%). A possible reason for this difference could be the enhanced spatial resolution of a BCI system with 64 electrode positions, compared to ReHand-BCI with 11. While a higher number of electrode positions has shown to increase %CA in BCI (Edlinger et al., 2015), an association between stroke patients’ control accuracy of a BCI system and upper limb motor recovery has not been proven. Therefore, further research is needed to ascertain its relevance for stroke rehabilitation.

Responses to the SUS questionnaire suggest that stroke patients in this study found the ReHand-BCI easy to use. The NASA-TLX results indicate that only moderate mental demand and effort were needed to use the system. This level of effort may have helped maintain patients’ motivation throughout the therapy, as it reflects that the task required for system control was neither easy nor too difficult. Patients evaluated their overall performance using the system as excellent. Therefore, the system could be improved by offering different levels of difficulty, which would help sustain their interest level during more extended treatment periods. Also, patients reported low frustration, coupled with low mental and temporal demands while using the ReHand-BCI. A system that is too demanding could make patients feel frustrated and potentially lead to the abandonment of the intervention.

### Limitations

The present work has the following limitations. First, a convenience sample of 10 patients is too low to accurately measure the differences in recovery, TMS, EEG, and HD measurements obtained after BCI and after conventional therapy. However, the sample size was adequate to perform an early clinical evaluation and had the advantage that each patient served as his or her own control. Moreover, the sample of this study was homogeneous regarding stroke-induced upper limb sequelae. Therefore, the results of this study support the hypothesis that the ReHand-BCI system has similar effects in stroke upper limb rehabilitation as conventional therapy and invite future clinical trials to assess this hypothesis.

Another limitation is the duration of the therapy since more than 1 month would be ideal for correctly assessing whether a BCI intervention improves the clinical and physiological outcomes of conventional therapy. Also, physiological measurements with a higher spatial resolution such as fMRI would provide additional insights into neuroplasticity-related mechanisms that could better elucidate the ReHand-BCI system’s efficacy for neurorehabilitation. Finally, the differences between therapies were calculated with the assumption that carryover effects were equal among groups. Carryover effects are an important limitation in crossover study designs; however, a washout period cannot be implemented without depriving stroke patients of valuable therapy sessions. Therefore, for ethical reasons, patients were provided upper limb rehabilitation therapy for the study duration, and no washout period was implemented. Despite these limitations, the findings support the hypothesis that ReHand-BCI is an effective approach to increase stroke patients’ recovery of upper-limb function.

## Conclusion

The present work assesses the feasibility of an intervention with a BCI system coupled to a robotic hand orthosis for stroke patients’ upper limb rehabilitation by comparing outcomes measured after a 12-session intervention using the ReHand-BCI with those obtained after the same number of sessions of conventional therapy. Also, system usability and subjective mental workload metrics were assessed after the BCI. This early clinical evaluation supports the hypothesis that the ReHand-BCI system can promote neuroplasticity and could be as effective as conventional therapy for upper limb recovery, but this still needs to be assessed in clinical trials. Also, future studies should consider interventions with a duration of more than 12 sessions to better assess gains in motor recovery. The measurement of physiological variables such as corticospinal tract integrity to complement clinical outcomes and the integration of user experience surveys that evaluate if the system has an adequate degree of difficulty for stroke patients will further elucidate the efficacy of BCI systems.

Finally, it has been suggested that increasing the frequency of interventions which include movement of patients’ paralyzed upper limbs, such as BCI therapies, could enhance stroke patients’ motor recovery (López-Larraz et al., 2018). However, the World Health Organization (WHO) has stated that the current rehabilitation workforce is insufficient in several parts of the world (Krug and Cieza, 2017). Therefore, the validation of BCI systems for stroke recovery is relevant as BCI systems could fill this gap by offering an additional tool to healthcare institutions, thereby increasing the number of patients that receive rehabilitation sessions or the frequency of these sessions, even in the early stages of stroke.

## Data Availability Statement

The datasets presented in this article are not readily available because of ethical reasons. Requests to access the datasets should be directed to the corresponding author.

## Ethics Statement

The studies involving human participants were reviewed and approved by the National Institute of Rehabilitation Ethics Committee. The patients/participants provided their written informed consent to participate in this study. Written informed consent was obtained from the individuals for the publication of any potentially identifiable images or data included in this article.

## Author Contributions

JC-N, RC-E, PC-M, JQ-F, and OA-C conceived and designed the study. JC-N, RC-E, MR-B, CH-A, MG-A, IH-S, and AM-P performed data collection. JC-N, RC-E, PC-M, JQ-F, and OA-C analyzed the data. JC-N, RC-E, PC-M, and OA-C drafted and edited the manuscript. JQ-F, MG-A, MR-B, CH-A, IH-S, and AM-P provided critical revisions. All authors have approved the final version of the manuscript submitted for publication.

## Conflict of Interest

The authors declare that the research was conducted in the absence of any commercial or financial relationships that could be construed as a potential conflict of interest.
